# Magnetic Resonance Imaging Enhancement Two Years Post Lumpectomy: An Unusual Timing for Fat Necrosis

**DOI:** 10.7759/cureus.14794

**Published:** 2021-05-01

**Authors:** Quan D Nguyen, Ahmad Kharsa

**Affiliations:** 1 Department of Radiology, University of Texas Medical Branch, Galveston, USA

**Keywords:** fat necrosis, enhancement, inflammation, malignancy, mri, breast

## Abstract

Breast fat necrosis is a common benign inflammatory process in the post-surgical or post-traumatic breast. It is often clinically silent with a wide spectrum of imaging findings that range from the characteristically benign to the suspiciously malignant. These characteristics pose a significant challenge for clinical providers to identify this common entity and distinguish it from more ominous diagnoses, especially in patients with a prior history of breast malignancies. We present a challenging case of fat necrosis with suspicious imaging features and an unusual timeline, occurring in the second year following lumpectomy. Radiologists’ awareness of this condition and its varying presentations is of critical importance to appropriately guide evaluation of suspicious magnetic resonance imaging enhancements.

## Introduction

Breast fat necrosis is a common benign inflammatory process in the post-surgical or post-traumatic breast. In fact, the five-year rate of fat necrosis after lumpectomy and radiation therapy in breast cancer patients is reported to be up to 40% [1]. Clinically, fat necrosis is usually silent, but may occasionally present with worrisome features for malignancy, including irregular masses, overlying skin retractions, ecchymosis, and skin thickness. Furthermore, the appearance of breast fat necrosis on different imaging studies can vary on a wide spectrum from the characteristically benign to the indeterminately malignant [1,2]. Combined, these characteristics pose a significant challenge for clinical providers to identify this common entity and distinguish it from more ominous diagnoses. Here, we present a challenging case of fat necrosis with suspicious imaging features and an unusual timeline, occurring in the second year following lumpectomy.

## Case presentation

**Figure 1:**
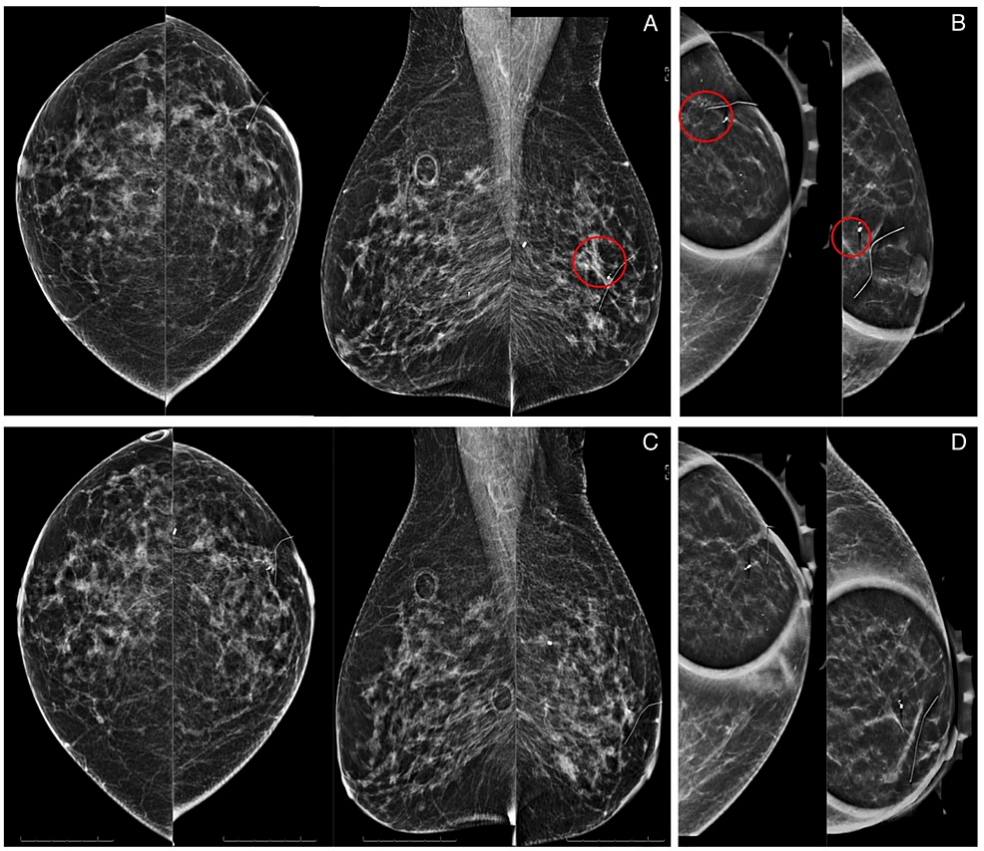
Surveillance mammograms for an asymptomatic 68-year-old patient with a history of breast malignancy who underwent lumpectomy. Craniocaudal and mediolateral oblique views (A) with spot magnification (B) of the bilateral breasts. Left breast shows focal asymmetry and associated calcifications at the lumpectomy bed (red circle). Craniocaudal and mediolateral oblique views (C) with spot magnification (D) from a prior mammogram two years earlier reveals no suspicious masses, calcifications, or other abnormalities.

A 68-year-old woman with a history of left breast ductal carcinoma in-situ status post left lumpectomy two years ago presented for her annual breast imaging surveillance. She denied any history of recent breast trauma, new breast lumps, breast discharge, or weight change. On physical examination, no dominant masses were appreciated in either breast bilaterally. Surveillance mammography revealed focal asymmetry and associated calcifications in the lumpectomy bed of the left breast (Figure 1).

**Figure 2:**
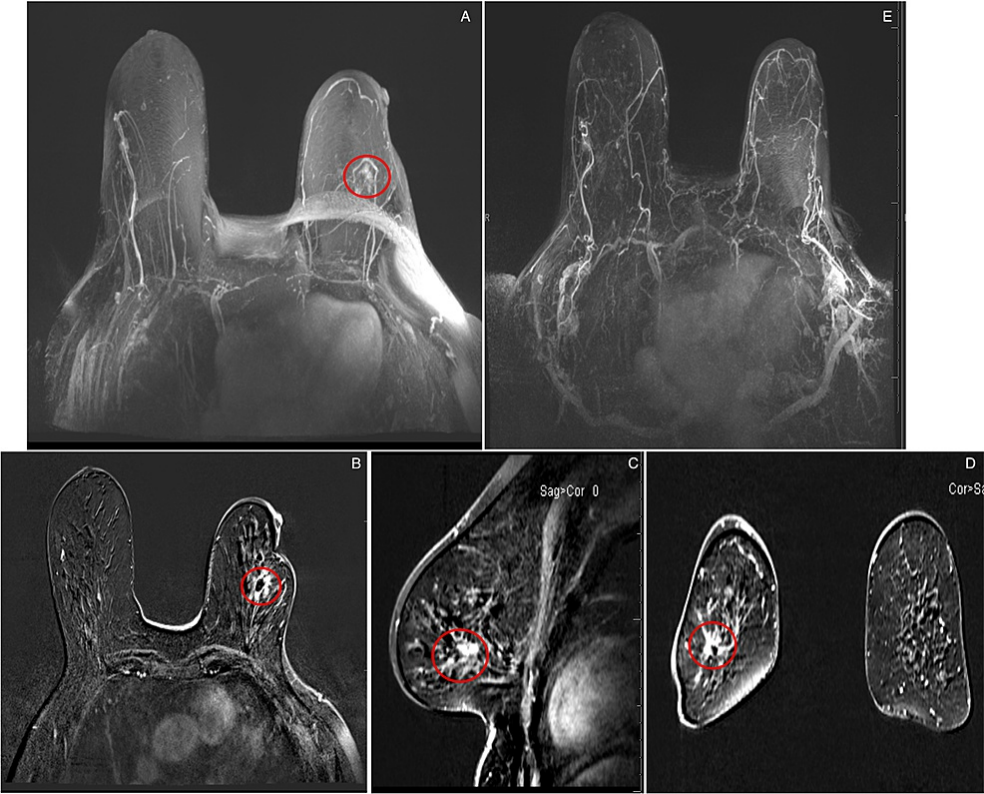
Surveillance MRIs for an asymptomatic 68-year-old patient with a history of breast malignancy who underwent lumpectomy. (A) Axial T1-weighted maximum-intensity-projection contrast-enhanced image shows non-mass enhancement (red circle) at the lumpectomy bed surrounding the fat necrosis. Axial (B), sagittal (C), and coronal (D) T1-weighted maximum-intensity-projection contrast-enhanced with subtraction image shows non-mass enhancement (red circle), measuring 26 × 16 × 22 mm at the 3 o'clock, 4 cm from the nipple surrounding the fat necrosis. (E) Axial T1-weighted maximum-intensity-projection contrast-enhanced MRI performed one year ago shows no abnormal enhancement. MRI: magnetic resonance imaging

Subsequently, magnetic resonance imaging (MRI) of the bilateral breasts with contrast demonstrated a new non-mass enhancement with heterogeneous internal enhancement in the left breast, concerning for malignancy recurrence (Figure 2).

No evidence of suspicious masses, abnormal enhancement, or other abnormal findings was detected in the right breast. Given the concerning presence of thick and irregular non-mass enhancement on MRI, a tomosynthesis-guided biopsy of the left breast was performed, and pathology confirmed the findings of fat necrosis with chronic inflammation and fibrosis.

## Discussion

Regardless of the imaging modality used, breast fat necrosis can present with variable appearance post breast conservation therapy. This variability lends itself to the histological basis of fat necrosis at the time of imaging, including the amount of visible liquefied fat, the severity of the inflammatory reaction, and the degree of fibrosis [2,3]. On mammographic imaging, fat necrosis can present as lipid cysts, focal asymmetries, microcalcifications, or spiculated masses, depending on the degree of fibrosis [3]. When minimal fibrosis occurs, breast fat necrosis usually presents as a lipid cyst with a predictable pattern of evolution, starting with linear and curvilinear calcifications and advancing to central calcifications. However, breast fat necrosis calcifications can present with a more suspicious appearance, visualizing as branching or angular calcifications. Furthermore, the fibrotic reaction may replace all of the radiolucent necrotic fat, resulting in the appearance of a focal asymmetric density on mammography similar to our case [1,3].

Despite these mammographic findings, post-lumpectomy changes, including lumpectomy bed scarring and fat necrosis, and breast cancer recurrence are often hard to distinguish on mammography and breast sonography. Therefore, MRI is often utilized to elucidate this distinction based on appearance and patterns of enhancement. Most commonly, the MRI appearance of necrotic fat usually resembles a lipid cyst, characterized by an oval or round hypointense signal on T1-weighted fat-saturated MRI. In many cases, the extent of the surrounding inflammatory change and granulation tissue results in a thin rim of peripheral enhancement. When these features coupled with other suggestive signs of fat necrosis, including signal isointensity of necrotic fat to surrounding breast tissue, compatible history of surgery or trauma, and typical mammographic correlates, are present, confidence in the diagnosis of fat necrosis can be established and biopsy may be deferred [2,3].

Unfortunately, there will be cases where even MRI fails to confidently clear the picture of breast fat necrosis from cancer recurrence. Sometimes, surrounding fibrosis can manifest as architectural distortion with thick, irregular, and spiculated margins of enhancement. Furthermore, and depending on the degree of associated inflammation, fat necrosis can produce enhancement following intravenous injection of paramagnetic contrast, thereby mimicking malignancy in morphology and contrast kinetics. In such equivocal cases as with ours, a biopsy may be indicated to differentiate breast fat necrosis from malignancy [3,4].

Beyond the mentioned imaging findings, timing is usually of critical significance in the evaluation of high-risk patients with MRI enhancements. Most cases of fat necrosis present within weeks to months following breast conservation surgery, whereas malignancy recurrence tends to occur in the first one to five years following surgery [1]. Nevertheless, our case presents an unusual timeline for enhancement in the lumpectomy bed, occurring in the second year following lumpectomy. With this in mind, radiologists should be aware of the different presentations of breast fat necrosis to appropriately guide evaluation of suspicious MRI enhancements.

## Conclusions

Breast fat necrosis is a common yet benign entity in the post-surgical breast that is critical to distinguish from other malignant processes in breast cancer patients. Given its often challenging and atypical presentation, a high index of clinical suspicion coupled with a characteristic appearance on imaging are needed to guide diagnosis. Nevertheless, in radiographically equivocal cases, a biopsy is needed to definitively rule out malignancy.
